# Otitis Media: Long-Term Effect on Central Auditory Nervous System

**DOI:** 10.1155/2019/8930904

**Published:** 2019-03-28

**Authors:** Maria Francisca Colella-Santos, Caroline Donadon, Milaine Dominici Sanfins, Leticia Reis Borges

**Affiliations:** ^1^Department of Human Development and Rehabilitation (DDHR), School of Medical Sciences, State University of Campinas (FCM/UNICAMP), Rua Tessália Vieira de Camargo 126, Cidade Universitária “Zeferino Vaz”, 13083-887 Campinas, SP, Brazil; ^2^Child and Adolescent Health Program, Center for Investigation in Pediatrics, School of Medical Sciences, State University of Campinas (FCM/UNICAMP), Tessália Vieira de Camargo 126, 13083-887 Campinas, SP, Brazil

## Abstract

**Objectives:**

To analyze the central auditory nervous system function through behavioral and electrophysiological tests in children with a history of otitis media and subsequent bilateral tubes placement surgery.

**Methods:**

The participants were divided into two groups between eight and 14 years old: control group (CG) consisted of 40 children with no history of otitis media; experimental group (EG) consisted of 50 children with documented history of otitis media and undertook a surgery for bilateral tubes placement. All children completed audiological evaluation (audiometry, speech audiometry, and immittance audiometry), behavioral evaluation (tests: dichotic digits, synthetic sentence identification with ipsilateral competing message, gaps-in-noise, frequency pattern), and electrophysiological evaluation (Auditory Brainstem Response, ABR, Frequency Following Response, FFR (verbal), and Long Latency Auditory Evoked Potential, LLAEP).

**Results:**

The EG group showed significantly poorer performance (p<0.001) than the CG for all auditory abilities studied. The results revealed significant latency delays and reduced amplitude (p<0.05) of waves III and V for ABR; significant latency delay was seen of potentials P2, N2, and P300 for LLAEP; significant latency delays and reduced amplitude (p<0.05) were observed for FFR in children with a history of otitis media.

**Conclusion:**

The results demonstrate negative effect of otitis media in the auditory abilities and electrophysiological measures in children with a history of otitis media.

## 1. Introduction

Secretory otitis media (SOM) is a clinical entity characterized by the presence of effusion in the middle ear, without perforation in the eardrum and acute infectious process for a period of three months. It is common in children between three and nine years old. The main symptom is a hearing loss, that is usually noted by parents or teachers, due to lack of attention and interest, request for repetition of the message several times and poor performance in school. The etiology is multifactorial and the highest incidence is caused by eustachian tube dysfunction and infections of the upper airways of allergic, viral, or infectious origin. With the advancement of the age, the maturity of the immunological system is completed, as well as the growth of the auditory tube, which decreases the occurrence of the disease [1].

The diagnosis is done by otoscopy and confirmed by audiological evaluation. It is possible to visualize by otoscopy and, frequently, a retracted eardrum with decreased mobility, opaque appearance, and abnormal color. In the audiologic evaluation, the diagnosis is a mild to moderate conductive hearing loss, usually bilateral, with a type B tympanometric curve. The hearing loss is fluctuating, temporary, and asymmetric [1].

The management of treatment could be clinical or surgical and depends on the middle ear conditions and a clinical history [2]. The most common treatment used for middle ear infection is a tympanostomy with tube placement insertion to drain the fluid in the middle ear and recover the hearing levels. However, children with SOM can show deficits in binaural hearing and auditory abilities even years after the otitis media has healed and pure-tone thresholds have returned to normal [3].

The central auditory processing (CAP) battery evaluates the effectiveness of the central nervous system's ability to process changing acoustic stimuli [4]. Currently, both behavioral and electrophysiological techniques are recommended to evaluate the processing of auditory information, in order to obtain more details regarding the functioning of the Central Auditory Nervous System (CANS), to perform a more precise diagnosis and to delineate the prognosis and intervention [5]. The behavioral evaluation of CAP allows the assessment of several auditory abilities and auditory evoked potentials tests such as Auditory Brainstem Response with click, Frequency Following Response (FFR) and Late Auditory Evoked Potentials (N1-P2-N2 complex and P300). These assessments enable us to have more information about the functioning of CANS through the extraction of signals that directly represent the brain activity in the auditory pathway, from the auditory nerve to the cortex in response to an auditory stimulus [6, 7].

When children are deprived of normal auditory input early in life, they can face CANS changes and diminished perceptual sensitivity to process auditory information later in life [3, 8]. Recent studies in human and animal have shown that sensory deprivation during development leads to long-lasting cellular deficits in auditory cortex and diminished behavioral performance [9, 10].

Therefore, the main purpose of this study was to analyze the long-term effect of otitis media in the peripheral and central auditory system, through behavioral and electrophysiological tests, in children with a documented history of SOM and a bilateral tubes placement insertion in the first six years of life.

## 2. Method

### 2.1. Study Design

This is a prospective cross-sectional study conducted at the Laboratory of Audiology from Department of Human Development and Rehabilitation/School of Medical Sciences from the State University of Campinas (Unicamp/Brazil), after its approval by the Ethics Committee (protocol 889074). Written informed consent was obtained for all participants.

### 2.2. Study Subjects

A total of 90 children, aged from 8 to 16 years old (mean 10.98 years old / 45 boys and 45 girls) from public school, participated.

The participants were divided into two groups: (i) the control group (CG) consisted of 40 children (17 boys and 23 girls, mean age of 10.7 years) with no history of otitis media and (ii) the experimental group (EG) consisted of 50 children (28 boys and 22 girls, mean age of 11.2 years) with a documented history of bilateral SOM in their first six years of life and with bilateral tympanostomy tube insertion.

The CG was recruited by the researcher at the state school and a questionnaire was filled by the parents about the child's health history. The EG was selected from the medical records at the State Hospital between 2000 and 2009 by the researchers.

The inclusion criteria for CG were as follows:

- Age between 8 and 16 years old
- Right handed
- Normal otoscopy bilaterally
- Hearing levels bilaterally within normal limits at the time of assessment (pure-tone audiometry thresholds below 20 dBHL at 250 to 8000 Hz) [11]
- Normal middle ear function (Type A) defined as a peak compliance within 0.3 to 1.3 mmhos and peak pressure within −100 to +20 daPa with the presence of ipsilateral and contralateral acoustic reflexes bilaterally between 70 and 100 dB for 500Hz, 1, 2, 3 and 4KHz [12]
- Typical development: good performance at school and language development, absence of attention disorder and auditory and respiratory complains

The inclusion criteria for EG were as follows:

- Age between 8 and 16 years old
- Right handed
- Normal otoscopy bilaterally
- Hearing levels bilaterally within normal limits at the time of assessment (pure-tone audiometry thresholds below 20 dBHL at 250 to 8000 Hz) [11]
- Normal middle ear function (Type A) defined as a peak compliance within 0.3 to 1.3 mmhos and peak pressure within −100 to +20 dPa with the presence of ipsilateral and contralateral acoustic reflexes bilaterally between 70 and 100 dB for 500Hz, 1, 2, 3 and 4KHz [12]
- Documented history of three episodes of SOM and only one set of bilateral tympanostomy tubes placement surgery in the first six years of life
- Absence of middle ear infections for the last 12 months until the date of the evaluation

Children with behavioral or neurological disorders and/or genetic syndromes, including those using psychoactive medication or attending speech therapy, were excluded from the sample.

### 2.3. Study Procedures

The protocol was composed based on three stages: hearing assessment, behavioral evaluation of central auditory processing, and electrophysiological evaluation.

For audiologic evaluation and CAP assessment, the audiometer AC-40-Interacoustics, TDH 39P headphones, and a Dell computer were used. In the electrophysiological evaluation, the equipment used was Biologic Navigator Pro-Natus. Immitanciometry was performed using the Interacoustics 235h. All equipment was calibrated according to ISO-389 and IEC-645 standards.

#### 2.3.1. Hearing Assessment

The parents were interviewed to obtain more information such as otological history and school performance. Next the hearing thresholds were assessed from 250 to 8000 Hz. Subsequently, speech recognition was assessed at 40dB HL using a list of 25 monosyllabic words from Portuguese in each ear, with a percentage of correct answers greater than 88% [13].

The tympanometry was obtained with the 226Hz probe. The contralateral and ipsilateral acoustic reflexes were performed in the frequencies of 500, 1000, 2000, 3000, and 4000Hz.

#### 2.3.2. Behavioral Evaluation of Central Auditory Processing

The tests were performed in one 45-minute session in a soundproof condition. The tests applied were dichotic digits (DD), synthetic sentence identification (SSI), gaps-in-noise (GIN), and frequency pattern test (FPT) [14–16].

*Dichotic Digits (DD).* The DD test developed in Brazil consists of four presentations of a list of two-syllable digits in Brazilian Portuguese, in which four different digits are presented simultaneously, two in each ear. The list contains 40 randomly arranged pairs of digits presented at 50 dB HL. The digits used to form the list are the numbers four, five, seven, eight, and nine. The participants were instructed to hear two numbers in each ear and repeat all the numbers they have heard. The order did not matter. The dichotic digit test verifies binaural integration ability [14].

*Synthetic Sentence Identification (SSI).* The SSI test consists of the presentation of ten Brazilian Portuguese synthetic sentences in the presence of competitive children's story, in the same ear, through the signal-to-noise ratios 0, -10, and -15. The intensity of sentence presentation was 40 dB HL. The task of the subject was to listen to the sentence and point it in the frame. The ability analyzed in this test was figure-ground [14]. 

*Frequency Pattern Test (FPT).* The FPT test is composed of three 150 msec tones and 200 msec intertone intervals presented at 50dBHL. The tones in each triplet are combinations of two sinusoids, 880 Hz and 1122 Hz, which are designated as low frequency (L) and a high frequency (H), respectively. Thus, there are six possible combinations of the three-tone sequence (LLH, LHL, LHH, HLH, HLL, and HHL). The subjects were instructed that they would hear sets of three consecutive tones that varied in pitch. The task of the subject was to repeat by humming and verbalizing the tonal pattern with the frequency patterns (e.g., high-low-high, low-low-high). The FPT test verifies temporal ordering ability [15]. 

*Gaps-in-Noise (GIN).* The GIN test consists of a series of 6-second segments of broad-band noise with 0 to 3 gaps embedded within each segment presented at 50 dB HL. The gaps vary in duration from 2 msec to 20 msec. The approximate gap-detection threshold is defined as the shortest gap duration which is correctly identified at least four out of six times. The participants were instructed to indicate each time they perceived a gap. The GIN test measures temporal resolution ability [16].

#### 2.3.3. Electrophysiological Evaluation

It was performed in a 60-minute session in a sound proofed and electrically shielded room. Before the beginning of the collection, the skin of each subject was cleaned in the places where the electrodes were fixed through an abrasive paste. Afterwards, the electrodes were placed with an electrolytic paste and with the aid of an adhesive tape the impedance was kept below 3kΩ and the interelectrode impedance less than 2kΩ.

During the evaluation, the subjects were instructed to keep their eyes closed in order to avoid artifacts. In 50% of the patients the assessment was initiated by the right ear, while the remaining 50% by the left ear. All electrophysiological assessments were performed monoaurally.

The electrophysiological evaluation was composed by three phases in the order below. The tests started on the right ear in 50% of the participants and in the left in the others 50%.

- Auditory Brainstem Response with click stimulus (nonverbal)
- Frequency Following Response – FFR (verbal)
- Late Auditory Evoked Potentials with tone burst stimulus (nonverbal)

(a) Auditory Brainstem Response with click (nonverbal): the electrodes were positioned according to the 10-20 system [17]. The stimuli were recorded with the active electrode at the vertex (Cz), the reference electrode at the ipsilateral mastoid, and the ground at the contralateral mastoid. This procedure allows verifying the integrity of the auditory pathway up to the brainstem area.

(b) Frequency Following Response (FFR): the test was performed with the same electrodes positioned for click ABR. The response was elicited using a 40 ms synthetic speech syllable /da/, provided by the BioMARK software and recorded by the Biologic Navigator Pro (Natus Medical). The stimulus consists of the consonant /d/ (transient portion or onset) and the short vowel /a/ (sustained portion or following frequency response). Two traces were performed twice with 3000 stimuli and free of artifacts. Subsequently, the responses were added giving rise to a third wave composed by 6000 stimuli. In the present study the FFR evaluation was performed by time domain analysis. VA complex measures (slope, related to the temporal synchronization of the response generators and area, related to the activity that contributes to wave generation) were also performed [18]. The FFR is elicited by verbal sounds that allow the analysis of the functional integrity of the auditory pathway through the information of the processing of short sounds (consonant) and the melodic contours (vowel) that are fundamental for a good communication [19–21].

The parameters used for ABR and FFR are described in Table 1. 

(c) Late auditory evoked potentials with tone burst stimulus (nonverbal) were recorded with the active electrode positioned on the vertex (Cz), the reference electrodes on the right (M2) or left (M1) mastoids and the ground electrode at the Fz position, according to the 10–20 system [17]. The right and left ears were assessed separately. The equipment was a 2-channel and a band pass filter of 1–30 Hz was used. The elicitor stimulus was delivered monoaurally through insert earphones at 75 dB HL. The infrequent target stimulus was a 2 kHz tone burst presented randomly with a probability of 20% and the frequent stimulus (nontarget) was a 1 kHz tone burst presented with 80% probability (oddball paradigm). The stimulus rate was one stimulus per second, with a total of 300 sweeps. A 533 msec time window was used and the analysis was based on the numerical values of the latencies (msec) and amplitudes (*μ*V). The P300 was identified as an infrequent stimulus after the complex N1, P2, and N2 (frequent stimuli). The analysis of the potentials was performed considering the values of latency and amplitude. The participants were instructed to mentally count the infrequent target tone, with the examiner verifying the task performance by asking them how many infrequent targets were counted. The ones who perceived more than 90% of infrequent stimuli were included in the research (see Table 2).

The latencies and amplitudes values of ABR, FFR and Late Auditory Evoked Potentials were viewed and marked manually by two blinded audiologists to avoid influence on the results. When there was difference in the marking, a third blinded audiologist analyzed the results and remained the mark that coincided with two equal analyses.

#### 2.3.4. Statistical Analyses

The groups were compared using ANOVA, for ABR, FFR and Late Auditory Evoked Potentials responses. The Wilcoxon-Mann-Whitney test was used for CAP responses. The gender and side were included in both models as a fixed effect, as well as their interactions. When the interaction effect between side and group was considered significant (p <0.05), the ears and gender were analyzed separately.

To test the homogeneity of the contingency tables, Pearson's Chi-square test was applied, setting the significance level of 0.05.

The statistical analyses were made through the software R-project (https://www.r-project.org).

## 3. Results

The distribution of the sample considering male and female gender and age group can be observed in Table 3. Analyzing the distribution between the control and experimental groups, considering the male and female gender (p value = 0.19) and the age group (p value = 0.455), it was verified that the sample is homogeneous.

Table 4 demonstrated no statistical difference between groups for hearing thresholds from 250 to 8000 Hz at the time of assessment. All the hearing thresholds were below 15 dB bilaterally for both groups.

### 3.1. Behavioral Central Auditory Processing

#### 3.1.1. Description of Results

Table 5 shows the results of behavioral evaluation of central auditory processing, comparing the responses between the CG and EG. When comparing the results, considering the right and left ears, it was verified that there was a statistically significant difference, in the EG, in the Digits Dichotic (p value = 0.001) and GIN (p value = 0.004) tests and the left ear had lower performance. In the other tests, the results of the two ears were combined for the other analyses. No significant difference was seen for gender in the behavioral tests. It was observed in the EG a lower performance than CG in the mean responses for the DD test of approximately 5% in both ears, 9.6% for the FPT (humming) and 30% (naming) and 8% for the SSI test. In the GIN test, the higher threshold obtained, the worse the test performance. There was a statistically significant difference for the gap-detection threshold between the studied groups, being the highest threshold obtained in the EG when compared to CG.

### 3.2. Electrophysiological Responses

#### 3.2.1. Description of Results

In the analysis of the ears for the ABR click, FFR and Late Auditory Evoked Potentials tests, no difference was observed between groups, in the measures of latencies and amplitudes. For this reason, the data of the two ears were combined in the other analyses. Considering the analyses for gender in the ABR, FFR and Late Auditory Evoked Potentials tests no significant differences were observed, only for slope VA (p=0,021).

**Figure 1:**
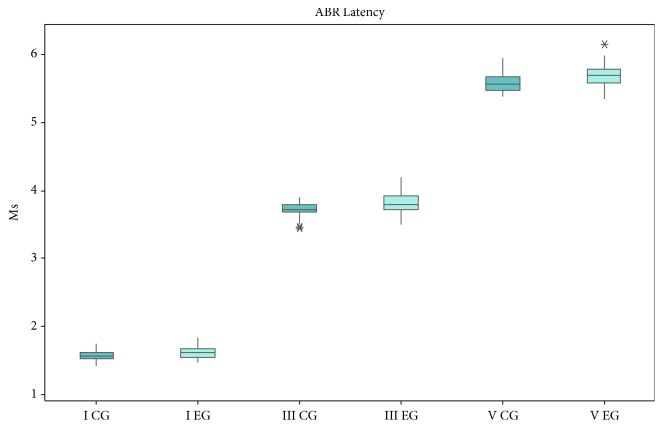
Box plots showing the median, interquartile, and range of latency (ms) of ABR for both control and EG groups.

The Auditory Brainstem Response with click stimulus measures showed a significant increase for latencies and decrease for amplitudes of waves III (0.1ms and 0.06*μ*V) and V (0.1ms and 0.05*μ*V) in the EG (see Table 6 and Figure 1).

**Figure 2:**
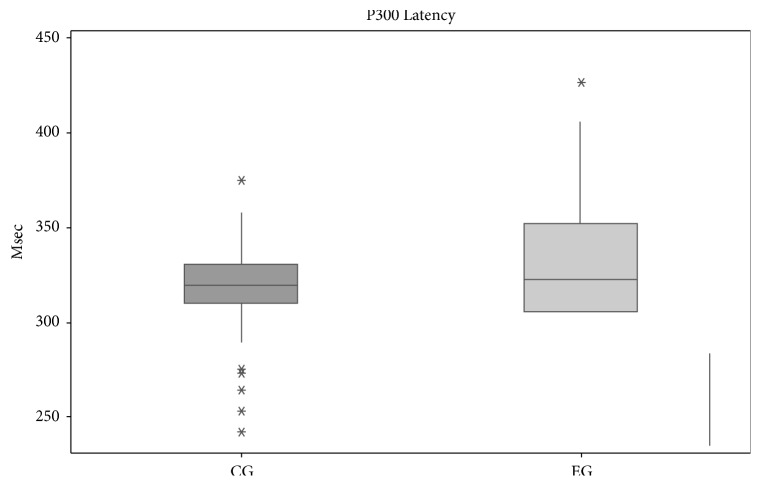
Box plots showing the median, interquartile, and range of latency (msec) of P300 for both control and EG groups.

The analysis of the Long Latency Evoked Potential between the control and experimental groups showed a statistically significant difference of P2, N2, and P300. The potential P2 had increased 9.21ms, N2 16.5ms, and P300 13.41ms in the EG compared to CG (see Table 7 and Figure 2).

**Figure 3:**
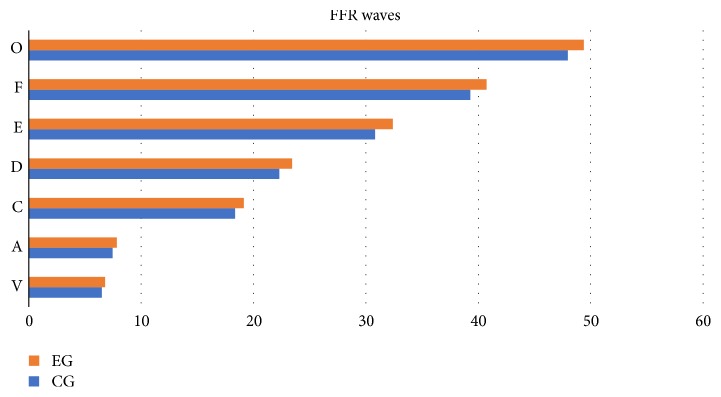
Latency (ms) values of FFR waves between groups.

For the FFR, it was verified that children from the EG presented an increase in latency values of all FFR components (V, A, C, D, E, F, and O waves) associated with a decrease in slope VA, in the female gender, comparing to CG (see Table 8 and Figure 3).

## 4. Discussion

This study was carried out with the purpose of analyzing the functioning of CANS in children with a history of bilateral SOM in the first six years of life with tympanotomy surgery for bilateral insertion of ventilation tubes.

Analyzing the mean responses of the CG and EG based on frequencies from 250Hz to 8KHz, both groups had equal hearing thresholds at the moment of the evaluation. Thus, in the EG it was found that the SOM did not cause a long-term negative effect in the peripheral system until the VIII cranial pair. The structures, mainly of the middle ear, recovered after the end of the disease. The comparative analysis of the hearing thresholds between the groups was important to show that the peripheral portion of the auditory system, probably, did not interfere in the responses of the behavioral evaluations of the CAP and electrophysiological measures.

### 4.1. Behavioral Central Auditory Processing

In the analysis of behavioral CAP responses, the EG showed a significant difference when compared to the CG. Thus, the children from EG could have difficulty processing the speech perception in the presence of background noise and combining auditory inputs from the two ears, in particular the integration of subtle timing, level, and spectral differences in the signals.

Our findings corroborate with the literature that studied the influence of OM in children and verified worse performance in auditory abilities [22, 23]. Borges et al. (2013) studied the influence of OM in children with different social-economic backgrounds and observed lower performance for DD and GIN. The authors concluded that the history of OM may change the central auditory function regardless of the socioeconomic status of the children.

### 4.2. Electrophysiological Evaluation

The results revealed significant latency delays and reduced amplitude of waves III and V for ABR and for FFR in children with a history of otitis media. The potentials P2, N2, and P300 also showed significant latency delays in children from EG. An increase in latency for N1 would not have been expected since N1 represents acoustic perception.

Regarding ABR, several studies have also described difference in latency and amplitude values in children with a history of OM [24, 25], but few studies have been found in the literature associating OM with long latency auditory evoked potentials.

Maruthy and Mannarukrishnaiah [8] evaluated the cortical potentials in children with early onset of OM and found an increase in the latencies of all components of the long latency auditory evoked potentials when compared to their normal peers. However, Shaffer [26] analyzed the long latency auditory evoked potentials responses in three OM conditions: few episodes, significant history, and active disease and observed an increase in N1 and P2 potentials only in the active OM group. In addition, the presence of P300 was not identified in any group. The author justified the absence due to short window time (500 ms). Our findings do not corroborate the results of this research, which may be due to the different methodology used such as the age of children and the window time.

For FFR, few studies have been found. El-Kabarity et al. [27] investigated children with bilateral SOM of recent onset and long duration. Fifty-five children between five and 11 years of age were divided into two groups: group I (25 children with long-term SOM) and group II (30 children with SOM with recent onset). Analysis of the results showed that FFR responses were statistically significant in the onset (wave V and A) and offset (wave O) portions in conjunction with the reduced values of the VA complex, more specifically, the slope VA, when compared to the responses of the group I in relation to group II. A recent research study by Sanfins et al. [28] studied FFR responses in two groups: (i) 30 children and adolescents with a history of SOM in the first years of life and (ii) 30 children and adolescents with normal hearing and typical development. The authors observed increased latency values in all components of FFR in children with a history of otitis media when compared to their healthy peers. Our study corroborates the research study cited above and has shown that the FFR seems to play an important role in the identification of auditory impairment in cases of history of otitis media.

Thus, our results demonstrated the negative effects of SOM in children, related to the maturation and functioning of the auditory pathways.

### 4.3. The Impact of Secretory Otitis Media in the Central Auditory Nervous System

The lower results obtained in the EG in both CAP and electrophysiological behavioral tests may have been due to the fact that recurrent SOM episodes caused, in the acute phase of the disease, an auditory sensorial deprivation, fluctuating, and often asymmetric hearing loss, in a critical period for the child's development.

As a consequence, the CANS received inconsistent, incomplete, and often different auditory information, considering the right and left ears, for an extended time, once the time between clinical treatments and the decision to perform the surgery can be long. Studies have shown that fluids remaining in an acute episode of OM remain in the middle ear for three to 12 months, and in 10 to 30% of children, the fluid remains for two to three months. Thus, a child who had three to four SOM episodes may have twelve months of conductive hearing loss at a time considered critical for their development and learning [29, 30].

Another consequence of these unfavorable conditions of stimulation may be a maturational delay in the structures of the CANS, a decrease in the number of stimulated nerve fibers and transmission. These changes in the CANS can interfere in the efficiency of the analysis and interpretation of the auditory stimuli, mainly related to the auditory abilities of figure background, ordering and temporal resolution which is fundamental for the development of speech, language and school performance.

The negative effects of otitis media in the measures of long latency auditory evoked potentials and FFR in the present study lead us to hypothesize that auditory pathway is affected from the brainstem level to the cortical level.

Thus, the effective diagnosis and medical treatment are essential. The earlier intervention in cases of otitis media can avoid the length of time of auditory fluctuation and minimize the effects caused by the fluid in the middle ear in the development of the auditory abilities. Also, it is important to refer all children who had a history of otitis media in childhood to an auditory evaluation once we observed that these individuals may have a risk to have a Central Auditory Processing Disorder.

It should be emphasized that more research regarding the effects of OM on behavioral and electrophysiological assessments should be made to guide parents and health professionals about the importance of hearing care, especially in the first years of life.

## 5. Conclusion

From the analysis of the results, the following was concluded.

There was a negative effect of otitis media on auditory abilities and electrophysiological measures in children with a history of otitis media. Concerning auditory abilities, the alterations observed were figure-background, ordering and temporal resolution. Electrophysiological tests revealed alteration from the brainstem to the cortical level.

## Figures and Tables

**Table 1:** Parameters of acquisition of the Click-ABR and FFR.

PARAMETERS	Click-ABR	FFR
Equipment	Biologic Navigator Pro	Biologic Navigator Pro
Stimulated Ear	RE and LE	RE and LE
Stimulus	Not verbal	Verbal
Type of the stimulus	Click	Speech
Duration of the stimulus	0.1 msec	40 msec
Polarity of the stimulus	Rarefaction	Alternate
Intensity of the stimulus	80 dBHL	80 dB SPL
Speed of the stimulus	19.3/sec	10.9 sec
Number of sweeps	2000	6000
Replicability	2 collections of 2000	2 collections of 3000
Filter	100-1500 Hz	100-2000 Hz
Window	10.66 msec	85.33 msec
Transducer	Insert (ER-3A; Natus Medical)	Insert (ER-3A; Natus Medical)

*Legend: RE*: Right Ear; *LE*: Left Ear; *msec*: milliseconds; *sec*: seconds; ABR: Auditory Brainstem Response; FFR: Frequency Following Response.

**Table 2:** Parameters of acquisition of the LLAEP with nonverbal stimulus.

PARAMETERS	NONVERBAL
Equipment	Biologic Navigator Pro
Stimulated Ear	RE and LE
Type of stimulus	Tone burst
Frequent stimulation	1000Hz (80%)
Infrequent Stimulus	2000Hz (20%)
Polarity of the stimulus	Alternate
Intensity of the stimulus	75 nHL
Speed of the stimulus	1.1/sec
Number of sweeps	300
Filter	1- 30 Hz
Window	533 msec
Transducer	Insert (ER-3A; Natus Medical)

*Legend: RE*: Right Ear; *LE*: Left Ear; *msec*: milliseconds; *sec*: seconds.

**Table 3:** Statistical analysis of the sample considering the gender and age between groups.

	CG	EG	p-valor
Number of children	40	50	
Age (Mean, years)	10.7	11.2	0.455
Gender (number)			
Male	17	28	0.19
Female	23	22	0.19

**Table 4:** Mean values of hearing thresholds in the right and left ears between control and experimental groups.

	250Hz	500Hz	1000Hz	2000Hz	3000Hz	4000Hz	6000Hz	8000Hz
RE-CG	8 dB	7.5 dB	6.5 dB	6 dB	4.5 dB	5.5 dB	12.5 dB	8.5 dB
RE-EG	8.3 dB	7.2 dB	5.5 dB	5 dB	4.4 dB	5 dB	12.2 dB	7.2 dB
p- value	0.589	0.200	0.361	0.687	0.358	0.324	0.950	0.198
LE- CG	8 dB	7 dB	5 dB	7,5 dB	4 dB	7 dB	8,8 dB	6,5 dB
LE-EG	8.8 dB	6.1 dB	4.4 dB	7 dB	5 dB	5 dB	10 dB	5 dB
p- value	0.892	0.301	0.486	0.154	0.909	0.150	0.926	0.672

Legend: RE: right ear; LE: left ear; CG: control group; EG: experimental group.

**Table 5:** Behavioral evaluation values of central auditory processing between control and experimental groups.

	CG			EG			
Test	N	Mean	SD	N	Mean	SD	P-value
*DD*							
RE	40	98.93%	1.86	50	95.40%	5.16	*<0.001*
LE	40	97.93%	4.15	50	92.55%	7.95	*<0.001*
*FPT *							
Humming	80*∗*	93.00%	12.4	100*∗*	83.40*∗*	18.5	*0.004*
Verbalizing	80*∗*	73.50%	21.2	100*∗*	42.7%	22.2	*<0.001*
*SSI*	80*∗*	67.5%	13.9	100*∗*	59.8%	16.9	*0.020*
*GIN*							
RE	40	4.65ms	1.00	50	6.22ms	1.40	*<0.001*
LE	40	4.72ms	1.06	50	6.56ms	1.52	*<0.001*

Legend: n: number; *∗*: number of ears; RE: right ear; LE: left ear; CG: control group; EG: experimental group; SD: standard deviation.

**Table 6:** Latency(ms) and amplitude((*µ*V)values of Click-ABR between groups.

	CG (n=80)		EG (n=100)		CGxEG
	Mean	SD	Mean	SD	p-value
*I*					
Latency	1.57	0.08	1.63	0.10	0.06
Amplitude	0.21	0.11	0.19	0.09	0.161
*III*					
Latency	3.71	0.11	3,81	0.15	*<0.001*
Amplitude	0.32	0.13	0.26	0.09	*0.002*
*V*					
Latency	5.59	0.14	5.69	0.17	*<0.001*
Amplitude	0.24	0.10	0.19	0.12	*0.008*
*I-III *					
Latency	2.14	0.11	2.19	0.15	0.124
*I-V*					
Latency	4.01	0.13	4.06	0.17	0.246
*III-V*					
Latency	1.87	0.11	1.88	0.11	0.977

Legend: CG: control group; EG: experimental group; SD: standard deviation.

**Table 7:** Latency (ms) and amplitude (*µ*V) values of Long Latency Auditory Evoked Potentials between groups.

	CG(n=80)		EG(n=100)		CG x EG
	Mean	SD	Mean	SD	p-value
*N1*					
Latency	107.7	23.19	108.9	19.38	0.864
amplitude	3.56	1.64	2.92	2.12	0.091
*P2*					
Latency	150.45	25.51	159.66	23.84	*0.011*
amplitude	3.47	1.38	3.71	2.73	0.288
*N2*					
Latency	202.67	31.87	219.17	35.51	*0.001*
amplitude	4.75	2.30	3.86	3.38	0.063
*P300*					
Latency	317.19	30.75	330.6	39.27	*0.008*
amplitude	5.52	2.13	5.42	2.42	0.794

Legend: CG: control group; EG: experimental group; SD: standard deviation.

**Table 8:** Latency (ms), Área VA (ms x *µ*v), and Slope VA (ms / *µ*v) values of FFR between groups.

Measure		Groups						
		CG			EG			
	Sex	N	Mean	SD	N	Mean	SD	p value
V		80*∗*	6.50	0.21	100*∗*	6.80	0.24	*<0.001∗*
A		80*∗*	7.47	0.34	100*∗*	7.85	0.32	*<0.001∗*
C		80*∗*	18.37	0.44	100*∗*	19.15	1.51	*<0.001∗*
D		80*∗*	22.29	0.57	100*∗*	23.44	1.94	*<0.001∗*
E		80*∗*	30.83	0.56	100*∗*	32.40	2.54	*<0.001∗*
F		80*∗*	39.29	0.52	100*∗*	40.75	2.66	*<0.001∗*
O		80*∗*	47.97	0.65	100*∗*	49.39	2.52	*<0.001∗*
Área VA (ms x *µ*v)		80*∗*	0.32	0.13	100*∗*	0.30	0.28	0.157
Slope VA (ms / *µ*v)		80*∗*	0.35	0.14	100*∗*	0.28	0.10	*<0.001∗*
	M	30*∗*	0.31	0.11	56*∗*	0.27	0.09	0.198
	F	50*∗*	0.39	0.14	44*∗*	0.29	0.10	*<0.001∗*

Legend: n: number *∗*: number of ears; SD: standard deviation; M: male; F: female; M+F: male and female; RE+LE: right ear and left ear; NA: not applicable.

## Data Availability

The data used to support the findings of this study are available from the corresponding author upon request.
